# Herpes Facialis1Read before the Central New York Medical Association, at its annual meeting, held at Rochester. October 20, 1896. 

**Published:** 1897-04

**Authors:** A. A. Young

**Affiliations:** Newark. N. Y. 22 East Miller Street


### HERPES FACIALIS.[1]

By A. A. YOUNG, M. D., Newark, N. Y.

IN CALLING your attention to herpes zoster, I shall confine what I have to say to that phase or subdivision of it known as herpes facialis. It matters not where the eruption may be located, so far as the eruption is concerned, for the excitant and the morbid conditions must be identical in each case.

One of the curious and constant features of the affection is for it to follow along the course of some nerve trunk, producing regional neuralgia, lasting for one or more days, previous to the appearance of the eruption over the affected part, and it is only with the appearance of the eruption that a diagnosis can be made. It is still debatable whether the disease is of microbic origin or not, but certainly the weight of evidence is in that direction. Just how bacteria enter the system, grow and multiply, producing pathological conditions which we call disease, is yet to be solved.

1. Read before the Central New York Medical Association, at its annual meeting, held at Rochester, October 20, 1896.

There are grave doubts of herpes being a dermal affection, though the anatomical lesions are largely found in the skin. It seems to be reasonably certain that the anatomical lesion is in some portion of the ganglionic nervous system and follows from such affected point along the course of a nerve which radiates either directly or indirectly from the affected ganglion.

The cell growths that form at the beginning and during the progress of this affection follow the course of certain blood-vessels and nerves dipping down to the subcutaneous areolar tissue, coming into contact with, and even encircling, the nerve tissue and causing in it changes in the neurilemma and the partial absorption, at least, of the white substance of Schwann, leaving the axis cylinder practically bare. With this condition, it is readily conjectured why neuralgia of most painful type exists, and why it exists prior to the eruption. Here we are led to ask, Is there not some method by which an early diagnosis can be made without waiting for the eruption? and when such diagnosis is made, cannot the disease be aborted? These questions are highly important, for the solution of them will aid us materially to avoid that which at times results disastrously under the most skilful treatment.

Leaving now the general discussion of herpes, the signs and symptoms of which all are doubtless more or less familiar, I pass to its localised form, or one of its subdivisions—namely, herpes facialis, or to still further localise herpes supraorbitalis.

Before proceeding farther, it is desirable at this point to turn our attention briefly to the anatomical relations of the nerve supply of the region about to be described. Beginning with the ophthalmic nerve, which practically arises in the casserian ganglion and is purely a sensory nerve, we reach the frontal nerve, which passes toward the eye, and at a point about midway between the apex and base of the orbit it divides again; one of its branches, the supraorbital, passes forward and upward through the supraorbital foramen and extending over the vault of cranium to near the occiput; in its passage it subdivides with the principal branch following near the course of the sagittal suture and parallel with it. The muscular branches of this nerve supply the corrugator supercilli, occipito-frontalis and orbicularis palpebrarum. It should also be remembered that the ophthalmic nerve supplies the eyeball, the lachrymal gland and the mucous lining of the eye. On account of the intimate relation which this nerve sustains to some of the most delicate structures of the body, to prevent pathological changes in them is most desirable, for a loss of function of a nerve means a corresponding loss of function of the parts which the nerve supplies.

The early diagnosis of zoster facialis is somewhat difficult to make, for in its early stages it may simulate brow ague, catarrh of the frontal sinus, iritis, and the like. The pain of herpes is constant and severe until the appearance of the eruption; the pain of brow ague is intermittent and corresponds to the chill, as observed in an uncomplicated case of ague, a disease, by the way, that is becoming rare. A differential diagnosis of inflammation of the frontal sinus can be made by the presence of a greater or less degree of nasal catarrh, but principally by the aid of the electric light applied near to or over the affected sinus; if inflammation exists with exudation the parts will appear more or less opaque, if not then translucent. Iritis may be differentiated by the varying character of the pain which passes more downward and along the sides of the nose, but principally by the appearance of the eye—namely, contracted pupil with more tolerance to light, the aqueous humor becomes yellow and serous, the iris losing its striated appearance, and the like.

In zoster the inflammatory condition of the eye begins rather with the mucous coat, which is supplied by the ophthalmic nerve and which is the part primarily involved by the disease under consideration. It has been observed that when the nasal nerve becomes involved in herpes the eye rarely escapes inflammatory action, but the inflammation affects the cornea rather than the iris; so the signs and symptoms of corneitis become most prominent, while those of iritis are partially obscured and become a secondary consideration.

I here present the history of a case that will give a clinical picture of the disease under consideration:

Miss S., a young lady about 18 years of age, called me on February 16, 1896. I found her in bed complaining of intense pain, beginning near the center of the orbit, extending slightly inward and upward and ending near the hair on the right side of the forehead. There was no swelling of the affected part, so far as could be discovered. Believing the case to be one of supraorbital neuralgia, induced by influenza, a corresponding treatment was ordered, but twenty-four hours later showed no signs of improvement; a slight puffiness of the skin over seat of pain now appeared with increased pain on pressure; there was a temperature of 100½°, with a pulse of 96; respiration not increased; no change in the line of treatment ordered. On my third visit the appearance of the patient was decidedly changed, the pain still marked, but not as intense as at my previous visits; the lids were swollen, there was marked conjunctivitis with chemosis; anesthesia of the cornea marked, the pupil responded sluggishly to light and shade, together with a moderate degree of corneitis. Passing from the region of the supraorbital foramen, slightly inward and upward, following the supraorbital nerve, was a well-marked eruption, which consisted of vesicles characteristic of herpes zoster as seen in other portions of the body. There could be no longer any doubt as to the character of the affection, although the location is rather an unusual one. The course of the vesicles followed along and above the path of the nasal nerve, hence we had some affection of the deeper structures of the eye; the external tunics were principally involved; the iris was dilated and but slight iritis could be detected; there was a rupture of three or four vesicles on the nose and forehead, which has left small scars. Knowing the seriousness of the affection, at least when the nasal branch of the nerve becomes involved, my efforts were chiefly directed toward lessening the severity and spread of the affection and confining it to the region of the supraorbital nerve and its branches, and in this I, or nature, was partially successful.

The internal treatment in this case consisted in the administration of acetanalid, hyoscyamia and morphia in varying quantities or proportion as her condition seemed to indicate. In the early stage fl. ext. jaborandi was given, the object of which was to stimulate the excretory organs to greater activity and, therefore, to eliminate some of the hypothetical bacilli, or some of the substances on which their life and growth depend, which is practically their destruction. The external treatment consisted in painting the affected portions with a combination of the tinctures of aconite and iodine—seven of the former and one of the latter—repeating several times daily, and after which cover the affected parts with "lintine," which is, as we all know, only absorbent cotton pressed into sheets. After the first external application the affection ceased to progress. Whether this is due to treatment or not I am not able to say, for I realise "one swallow does not make a spring." It may, however, indicate its approach. Her recovery was more rapid than I anticipated. She reported at my office in person about three weeks after the onset of the disease, eyelids still slightly swollen, but not painful, and of a brownish color; ptosis was marked; the vesicles had nearly all disappeared, except in her hair, near the vertex of her cranium; the pupil was still dilated to about four times its normal size; there was partial anesthesia of the conjunctiva and cornea, but not as marked as when the affection was active. She was now given a preparation of iron, strychnine and gelsemium. She was requested to report to me in one week, but failed to do so for a considerable time.

In the picture herewith given the external condition of the eye is shown as it existed immediately after healing of vesicles and at the time of beginning of treatment for its pathological condition. The dotted lines indicate the course of the eruption ; ptosis well marked ; pupil dilated to four times the size of its fellow and not responsive to light and shade ; conjunctiva congested and hyperesthetic ; general condition of patient otherwise good.

When seen again the swelling had disappeared, ptosis scarcely observable, slight cloudiness of cornea, pupil dilated to at least four times the size of its fellow and vision partially obstructed ; otherwise the condition of the patient was that of perfect health. For the space of six weeks longer she remained under my care for the abnormal condition of the eye, the treatment of which consisted in placing over the cornea an ophthalmic disc of gelatin containing 1-1000 grain of eserin sulph. The pupil would readily respond to the action of the drug and even become smaller than the pupil of the unaffected eye ; the pupil would, after a few hours, dilate again, but not to the extent it previously had been ; it is now less than double the size of its fellow and vision fairly good.

The indications are that the limit toward recovery in this case is now reached and that the eye is ever to remain in its present condition, with dilated pupil and slight opacity of cornea, as a result of inflammatory action. It is impossible to say what the result would have been had a correct diagnosis been earlier made ; but it is my belief, from observation, that the eye complications might have been avoided by early treatment along lines that were later followed in this particular case.

Of the external treatment of herpes by the combination of aconite and iodine, when applied early, most gratifying results have been obtained ; they certainly control the pain and also limit the number and extent of the vesicles. The excretory organs also need attention ; they need stimulation, and for this purpose preference is given to pilocarpin, pushed till the physiological effects are observed.

From past experience in the early stages of herpes it is inferred that the treatment herewith outlined will materially lessen its severity, if not practically abort it.

22 East Miller Street.

